# Organic and Perovskite Solar Cells with Printed Electrodes

**DOI:** 10.3390/polym18172037

**Published:** 2026-08-22

**Authors:** Kyungsik Kim, Yeong-Ho Kim, Jinho Lee, Soonil Hong, Jong-Hoon Lee

**Affiliations:** 1 Department of Physics, Department of Intelligent Semiconductor Engineering, Incheon National University, Incheon 22012, Republic of Korea; kyungsik9905@inu.ac.kr; 2 Department of Advanced Materials Engineering, Kyonggi University, Suwon 16227, Republic of Korea; ho5426@kyonggi.ac.kr; 3 Division of Advanced Materials, Korea Research Institute of Chemical Technology (KRICT), Daejeon 34114, Republic of Korea; 4 Advanced Materials and Chemical Engineering, University of Science & Technology (UST), Daejeon 34114, Republic of Korea

**Keywords:** organic solar cells, perovskite solar cells, polymer electrodes, carbon electrodes, printing technology

## Abstract

Organic solar cells (OSCs) and perovskite solar cells (PSCs) are emerging photovoltaic technologies owing to their high efficiency, low-cost processing, and diverse applications ranging from utility-scale power generation to small-scale electronics. A key advantage of OSCs and PSCs over traditional silicon-based solar cells is their solution-processability, which enables fabrication via cost-effective scalable printing technologies suitable for commercialization. In addition to organic and perovskite photoactive layers, other functional layers, including interfacial layers and electron and hole transport layers, can also be processed using solution-based printing techniques. However, the conventional architecture of these emerging photovoltaics relies on vacuum-based deposition processes for both oxide-based electrodes (e.g., indium tin oxide and fluorine tin oxide) and metallic top electrodes (e.g., Au, Ag, Cu, and Al), which contrasts with printing-based processing. The implementation of printing technologies for electrode fabrication is necessary to achieve low-cost production and flexible photovoltaic applications. Herein, we review printed electrodes—including metal electrodes, conductive polymers, and carbon-based materials—used to fabricate OSCs and PSCs.

## 1. Introduction

In accordance with global carbon neutrality policies aimed at mitigating the climate crisis, solar cells that convert inexhaustible solar energy into electricity have been steadily developed [1]. In recent years, third-generation solar cells, such as organic solar cells (OSCs) and perovskite solar cells (PSCs), have attracted significant attention for applications in building-integrated photovoltaics (BIPVs), vehicle-integrated photovoltaics (VIPVs), and indoor light-harvesting devices suitable for modern urban environments [2,3,4]. One of the key advantages of solar cells over other renewable energy technologies lies in their low barriers to entry, the absence of large components, and their potential for low-cost mass production based on semiconductor processes [5]. In particular, compared with conventional silicon-based solar cells, OSCs and PSCs are expected to enable lower-cost production owing to their low-temperature processing (≤150 °C) and their compatibility with solution-based printing processes [6,7]. However, high-efficiency devices are typically fabricated using spin-coating or vacuum deposition techniques rather than printing-based processes [8,9,10,11]. This reliance inherently limits their scalability toward high-throughput, large-area manufacturing.

In particular, for solution-based processes, a transition from the spin-coating method with precise control of film thickness and uniformity via rotational speed (rpm) to scalable high-throughput deposition techniques is needed. Printing technologies such as doctor-blade coating, slot-die coating, ink-jet printing, and screen printing have been widely adopted to enable effective scale-up to large-area devices and modules [12,13]. Since 2009, the F.C. Krebs group has utilized various printing technologies to scale OSCs from unit cells to large-area modules [14,15]. The deposition of photoactive layers and charge transport layers has primarily been carried out using roll-to-roll (R2R)-compatible slot-die coating and knife edge coating processes. In addition, several types of silver (Ag) paste have been adopted for large-area devices using screen printing methods [16]. In PSCs, the evolution from a mesoscopic titanium dioxide (TiO_2_) structure to a planar-type structure has facilitated the adoption of printing-based fabrication processes such as spray coating, blade coating, and slot-die coating [17,18,19]. D. Vak’s group first demonstrated printed PSCs, with the exception of the electrodes, by introducing slot-die coating and a gas-quenching process under ambient air conditions [19]. Currently, these printing technologies have expanded from unit-cell devices to large-area modules, with module power conversion efficiencies (PCEs) reaching 14.5% for OSCs and 21.1% for PSCs, as reported in the National Laboratory of the Rockies (NLR) chart [20,21,22].

With increasing PCEs of OSCs and PSCs, achieving cost competitiveness is just as crucial as ensuring high efficiency and long-term stability [23,24]. These solution-processable solar cells enable high-throughput and low-cost printing techniques, thereby reducing manufacturing costs compared with conventional vacuum-based processes. However, vacuum-deposited metal electrodes are still employed as top electrodes to achieve high efficiency and long-term stability, while indium tin oxide (ITO) or fluorine-doped tin oxide (FTO) is typically used as the bottom electrode [25,26]. Top electrodes such as Ag are high-cost materials whose cost is directly proportional to their usage, in contrast to the largely fixed cost of transparent bottom electrodes. Vacuum evaporation produces chamber-wall losses, while printing can reduce material waste by depositing the electrode only where it is required. However, unused ink, edge trimming, cleaning, and rejected devices must still be considered as fabrication losses [27,28,29]. For comparison, a 100 nm dense Ag film contains approximately 1.05 g m^−2^ of Ag, while a 15 μm printed Ag layer is 150 times thicker before accounting for paste solids, porosity, and patterned coverage. Therefore, meaningful cost reduction requires minimized Ag coverage, reduced dependence on ITO/FTO, and high manufacturing yield. At fixed material and process costs, increasing the yield from 50% to 90% reduces the cost per delivered watt by approximately 44%.

This review aims to discuss OSCs and PSCs based on printed electrodes, focusing on metallic pastes such as Ag paste, conductive polymers such as poly(3,4-ethylenedioxythiophene):polystyrene sulfonate (PEDOT:PSS), carbon-based electrodes, and composite electrodes. Rather than focusing on conventional laboratory-scale spin coating or high-vacuum deposition, we specifically emphasize materials and processing strategies that enable low-cost, continuous, and scalable manufacturing such as blade coating, slot-die coating, screen printing, and lamination methods. This review also examines the applicability of printing-based electrodes to both opaque devices and semitransparent solar cells.

## 2. Printable Electrodes for OSCs and PSCs

### 2.1. Metallic Electrodes

**Figure 1:**
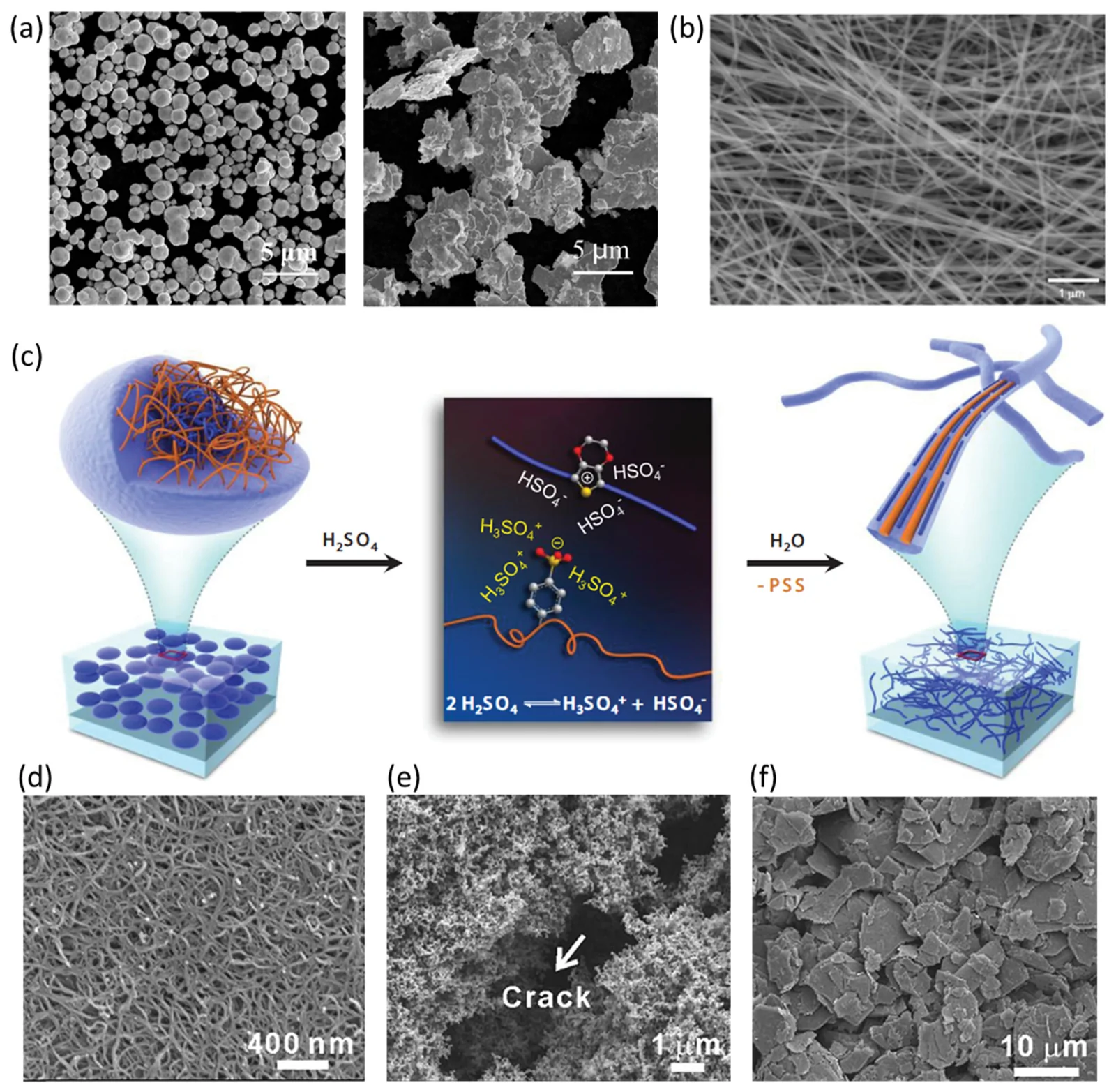
(**a**) SEM images of the spherical silver powder and flake silver powder. (**left**) Spherical silver powder. (**right**) Flake silver powder. Reproduced with permission from Ref. [32] Copyright 2022, MDPI. (**b**) SEM image of Ag nanowires. Very few small particles are present, indicating the high purity of the material following the separation process. Reproduced with permission from Ref. [45] Copyright 2007, American Chemical Society (**c**) Diagram of the structural rearrangement of PEDOT:PSS. The amorphous PEDOT:PSS grains (**left**) are reformed into crystalline PEDOT:PSS nanofibrils (**right**) via a charge-separated transition mechanism (**middle**) via a concentrated H2SO4 treatment. Reproduced with permission from Ref. [49] Copyright 2014, John Wiley & Sons Inc. SEM images of (**d**) multiwalled CNTs, (**e**) carbon black and (**f**) graphite. Reproduced with permission from Ref. [67] Copyright 2015, Royal Society of Chemistry.

As crystalline silicon solar cells have advanced toward mass production and cost reduction, screen-printed Ag paste has emerged as a key electrode material, replacing vacuum evaporation processes, which suffer from substantial material waste during electrode deposition [30,31]. Ag paste is prepared by homogeneously mixing Ag powder, glass frit, and an organic vehicle, followed by a three-roll milling process to ensure high dispersion and optimal rheological properties for screen printing (Figure 1a) [32]. As early Ag pastes were designed for high-temperature firing (~800 °C), they could naturally diffuse through the passivation layer. This ‘fire-through’ capability facilitated direct ohmic contact formation with the silicon substrate, bypassing separate etching or removal of the protective coating [30,31,32,33,34,35]. Since the 1990s, the demand for low-temperature Ag paste has intensified, primarily driven by the commercialization efforts of silicon heterojunction solar cells [36,37]. With the development of OSCs, low-temperature Ag pastes began to be developed to enable fabrication via R2R processes [15]. Since then, these materials have evolved into essential electrode solutions for next-generation photovoltaics, i.e., PSCs, which also require strict thermal budget management [38].

Compared with vacuum-deposited metal electrodes, printed Ag pastes generally exhibit greater thickness and surface roughness as these electrodes are formed from Ag particles and organic binders. These characteristics can increase contact resistance and potentially induce interfacial defects or shunting in thin-film photovoltaic devices. Nevertheless, optimized printed Ag electrodes can achieve low sheet resistance and device efficiencies comparable to those obtained using vacuum-deposited Ag. For example, rapid curing and sintering processes have enabled printed Ag-based OSCs to approach the photovoltaic performance of evaporated Ag reference devices. Therefore, although vacuum-deposited metals still provide advantages in terms of film uniformity and interfacial quality, printed Ag electrodes offer substantial benefits for scalable manufacturing by eliminating vacuum processing and enabling direct patterning with high material utilization.

Although Ag pastes have been widely adopted in industrial applications, their high cost has driven the development of alternative paste materials such as aluminum (Al) paste and copper (Cu) paste. Al paste is a fundamental material for forming the rear electrodes of crystalline silicon solar cells because it is much less expensive than Ag [39]. However, Al is readily oxidized in air, forming an insulating Al_2_O_3_ layer, which makes it difficult to achieve high-conductivity electrodes via low-temperature processes. Cu has an electrical conductivity nearly identical to that of Ag, and Cu paste has been considered the primary candidate for replacing Ag electrodes. Nevertheless, persistent oxidation challenges have confined its commercialization to the initial phase of rear-side metallization in crystalline silicon solar cells. To overcome these limitations, hybrid pastes in which Ag is coated onto Cu particles and alloy-based pastes have been developed [40,41,42]. As a transparent electrode, Ag nanowires (AgNWs) are one-dimensional metallic nanostructures that form a percolating conductive network on a substrate, offering high optical transparency and excellent electrical conductivity simultaneously [43,44]. AgNWs are typically synthesized via the polyol process, where AgNO_3_ is reduced in hot ethylene glycol and anisotropically grown into a one-dimensional wire structure through the selective capping of polyvinylpyrrolidone on specific crystal facets (Figure 1b) [45].

### 2.2. Polymer Electrodes

Since the 1970s, the application of doping strategies to polyacetylene has dramatically increased the conductivity, exceeding the order of magnitude reported by H. Shirakawa, A.G. MacDiarmid, and A.J. Heeger [46]. Following the initial discovery, conjugated polymers have undergone continuous advancement, resulting in conductive polymers that exhibit high conductivity up to 1000 S/cm [47,48]. Polyaniline (PANI), polypyrrole (PPy), and PEDOT:PSS are representative solution-processable conductive polymers. Among these conductive polymers, PEDOT:PSS is unique because its electrical, optical, and mechanical properties can be systematically tailored through chemical and structural engineering of the polymer network (Figure 1c) [49]. Commercial formulations are available over a broad range of conductivities and viscosities, allowing their adaptation to different printing techniques. In contrast, other conducting polymers, such as PANI and PPy, generally suffer from more limited solution processability, optical transparency, film-forming ability, or formulation stability. Therefore, despite continuing efforts to develop alternative conductive polymers, PEDOT:PSS remains the most practically established conductive polymer for printed photovoltaic electrodes. In particular, a strategy involving acid treatment has demonstrated conductivities approaching 4000 S/cm for PEDOT:PSS [49,50,51]. In addition, PEDOT:PSS enables intrinsically flexible and mechanically compliant electrodes, making it particularly attractive for ultrathin, stretchable, and wearable solar cell platforms [52,53].

Although PEDOT:PSS exhibits relatively high conductivity, its conductivity is often still insufficient for use as a standalone electrode, leading to its widespread application in semitransparent solar cells [54,55,56]. Depending on the device architecture, PEDOT:PSS can function as a hole transport layer (HTL), interfacial modifier, transparent front electrode, or transparent top electrode [57,58,59,60]. When used as an electrode, however, PEDOT:PSS must satisfy more demanding requirements than a conventional thin HTL; it must provide sufficient lateral conductivity, high optical transmittance, smooth morphology, a stable work function, good adhesion, and chemical compatibility with adjacent layers. Printing-based electrode applications remain uncommon even for PEDOT:PSS, the most conductive polymer electrode, and are even rarer for other conductive polymers such as PANI and PPy. Additionally, its high transparency and appropriate work function as an HTL enable its application as a tandem solar cell intermediate electrode, but uniform deposition over large areas presents a significant challenge [61,62,63,64].

### 2.3. Carbon Electrodes

Carbon-based electrodes have attracted considerable attention as sustainable alternatives to conventional metallic electrodes because they combine mechanical robustness, chemical stability, and low environmental impact with compatibility for solution-based fabrication processes [65,66]. Unlike vacuum-deposited metal electrodes, carbon-based conductive materials—including carbon nanotubes (CNTs), carbon black, graphene, and graphite composites—can be fabricated using low-temperature solution-processing techniques such as screen printing, spray coating, transfer lamination, brush painting, and aerosol-assisted deposition (Figure 1d–f) [67]. Early carbon electrodes were primarily based on naturally occurring graphite or amorphous activated carbon. The discovery of soccer-ball-shaped carbon molecules in 1985 opened up new possibilities for their use as electron transport layers (ETLs) and electrode materials in organic electronic devices [68]. In 1991, S. Iijima discovered CNTs, characterizing their hollow inner core and multilayered rolled graphene structure spanning a few nanometers in diameter [69]. In 2004, the revolutionary isolation of graphene from graphite using Scotch tape by K. Novoselov and A. K. Geim, which earned them the 2010 Nobel Prize, represented a milestone in the evolution of carbon electrodes [70].

CNTs are characterized by outstanding properties, such as high tensile stress and superior electrical and thermal properties, which allow them to replace thermally evaporated noble metals (Ag and Au) in solar cells [26]. CNTs are broadly classified into single-walled CNTs (SWCNTs), which consist of a single layer, and multiwalled CNTs (MWCNTs), which are composed of two or more layers [71,72,73]. Carbon black is a fine carbon powder featuring excellent electrical conductivity and cost-effectiveness and is widely utilized as a conductive additive or blending agent in carbon paste electrodes to facilitate efficient charge extraction in solar cells [74]. While pristine graphene exhibits exceptional electrical properties in its single-layer form, its practical application in devices remains challenging; hence, it is commonly utilized as an HTL in the form of reduced graphene oxide (rGO) or incorporated with other polymers for electrode applications [75,76,77,78]. To utilize these carbon electrodes in printing processes, they are formulated into a carbon paste, which is a composite of a polymer binder and organic solvents.

### 2.4. Hybrid Electrodes

Hybrid electrodes combine two or more conductive components to overcome the limitations of single-material electrodes [78,79,80]. This strategy is particularly important for OSCs and PSCs because no single printable electrode material simultaneously provides high conductivity, optical transparency, chemical compatibility, suitable energy-level alignment, mechanical flexibility, and low-cost scalability. Hybrid electrodes decouple these functions into different materials or layers. In a typical hybrid design, one component provides a chemically compatible interface with the photoactive or charge transport layer, while another component provides lateral current collection, transparency, or mechanical reinforcement [43,81]. Considering these aspects, hybrid electrodes should be approached as multifunctional contact systems rather than as simple mixtures of conductive materials. Hybrid electrodes that synergistically combine AgNWs, polymers, and carbon are under active investigation [79,80,81]. This approach aims to address the inherent drawbacks of individual components, including the high surface roughness and high cost of AgNWs, the limited electrical conductivity of carbon, and the moderate conductivity of polymers.

A representative example is the AgNW/PEDOT:PSS hybrid electrode, which features a structure where a conductive polymer such as PEDOT:PSS is thinly coated via spin-coating or slot-die coating over a randomly oriented AgNW network [79,80,81,82,83]. Compared with pristine PEDOT:PSS and AgNW electrodes, AgNW/PEDOT:PSS hybrid electrodes provide a more favorable balance between conductivity and optical transparency. The AgNW network forms highly conductive pathways for lateral current collection, thereby reducing the dependence on charge transport through the relatively less conductive PEDOT:PSS matrix. PEDOT:PSS, in turn, bridges AgNW junctions, reduces junction resistance, planarizes the rough nanowire network, and improves interfacial contact with adjacent functional layers. This complementary combination can reduce sheet resistance without requiring a thick PEDOT:PSS layer and therefore minimizes the corresponding optical loss. In addition, embedding AgNWs within PEDOT:PSS can improve electrode mechanical integrity and reduce the risk of direct nanowire contact with the thin photoactive layer. Similarly, carbon/PEDOT:PSS hybrid electrodes consist of a conductive PEDOT:PSS matrix that fills the spaces within CNT or two-dimensional graphene networks [65,84,85,86,87]. Furthermore, carbon/metal hybrid electrodes are among the most practical printed-electrode designs for large-area solar cells [88,89,90]. In this structure, carbon serves as the perovskite-compatible rear contact, while a metallic component provides low-resistance current collection. Rather than reassigning this concept to a single electrode material, the hybrid design separates the chemically sensitive vertical contact from the laterally conductive current-collecting pathway.

## 3. OSCs with Printed Electrodes

### 3.1. OSCs with Printed Metallic Electrodes

Printed Ag paste electrodes represent one of the most industrially viable approaches for replacing vacuum-deposited metal electrodes in OSCs because they directly leverage mature printing technologies already established in the electronics manufacturing industry. Their intrinsically high electrical conductivity and compatibility with high-throughput processes such as screen printing and flexographic printing make them particularly attractive for large-area and R2R photovoltaic production [91,92]. However, direct application of Ag pastes to OSCs is challenging because the thick and rough metallic layers can induce severe shunting, poor interfacial contact, and optical loss in thin organic active layers [93,94,95]. Recent studies have therefore focused on improving the morphology, reflectivity, conductivity, and interfacial compatibility of printed Ag electrodes to achieve efficient fully solution-processed OSCs.

**Figure 2:**
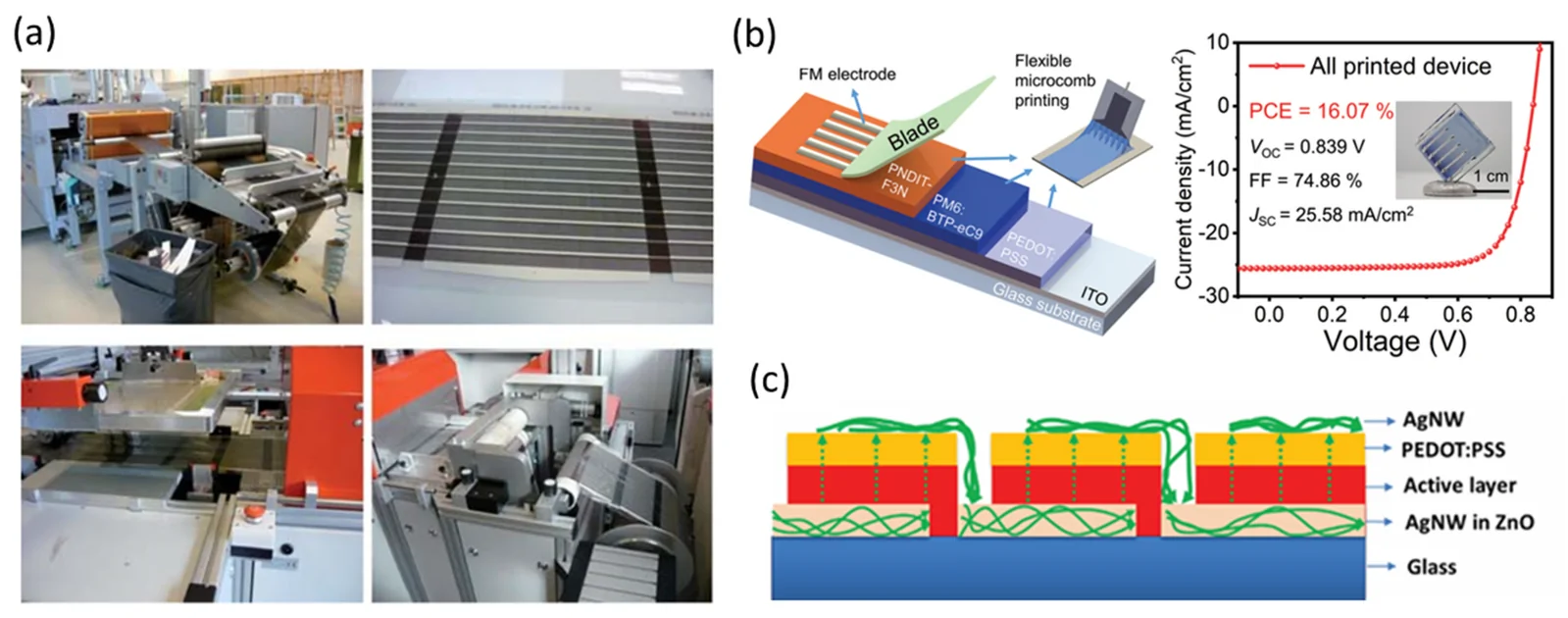
(**a**) Screen printing of silver using a Klemm line as seen from the un-winder (**top left**) and the same device type (12 × 18 mm^2^ stripes) after having printed the Ag (**top right**). Flat bed screen printing using a flat bed screen printer from Alraun (**bottom left**) showing 16 × 13 mm^2^ stripes on the re-winder (**bottom right**). Reproduced with permission from Ref. [16] Copyright 2010, The Royal Society of Chemistry. (**b**) All-printed OPVs: (**left**) device architecture and the schematic of fabrication process, (**right**) J–V curve of the all-printed champion device. Reproduced with permission from Ref. [98] Copyright 2023, John Wiley & Sons Inc. (**c**) Schematic illustration of fully-printed OSC modules with AgNW electrodes in ZnO. Reproduced with permission from Ref. [99] Copyright 2021, John Wiley & Sons Inc.

One major strategy involves the use of screen-printed Ag top electrodes for inverted OSCs [96]. The F.C. Krebs group led the way in utilizing screen-printed Ag pastes to form back electrodes in fully printed OSCs, as shown in Figure 2a [16]. Various Ag inks were evaluated using screen printing, and UV-curable ink showed the best PCE of 2.2% in OSC modules consisting of 16 serially connected subcells. Kim et al. demonstrated solution-based screen printing of Ag top electrodes on PEDOT:PSS layers using a high-viscosity Ag ink, achieving homogeneous and void-free electrode morphologies with a very low sheet resistance of approximately 0.06 Ω/sq and an average electrode thickness of ~15 µm [96]. The PEDOT:PSS layer also acted as a protective barrier, suppressing Ag diffusion and minimizing damage to the underlying organic layer during printing. Similarly, Chung et al. employed screen-printed Ag electrodes combined with near-infrared (NIR) rapid curing to replace thermally evaporated electrodes in fully solution-processed OSCs [97]. By optimizing the NIR irradiation conditions, the curing time of the Ag paste was dramatically reduced from 30 min to only 5 s while maintaining a low electrode resistance (~0.3 Ω) comparable to that of evaporated Ag. The optimized devices achieved PCEs of 2.90–2.97%, nearly identical to those of evaporated reference devices (~3.08%), while module-scale devices retained an efficiency of 2.62%. Importantly, these devices exhibited excellent scalability and stable operation over 500 h of storage under both N_2_ and ambient conditions.

In the case of Ag pastes, a relatively thick HTL is often required to prevent damage to the photoactive layer caused by the solvent, making it difficult to achieve high device efficiency. Recently, Liu et al. achieved a PCE of 16.07% in OSC devices fabricated using an indium–tin Field’s metal alloy ink (FM) through a blade coating process (Figure 2b) [98]. As a top electrode, this alloy has a low melting point of 62 °C and enables a printing process with nontoxic elements. By optimizing the thickness of the alloy ink, the coating parameters, such as the blade coating speed and substrate temperature, were controlled. Using a tungsten mask, the printed area of the alloy ink is defined as a length of approximately 3 mm. Although the feasibility of applying alloy inks to large-area devices has not yet been demonstrated, this result represents the highest reported efficiency among devices employing printing-based top electrodes. Another important advancement is the realization of fully printed OSC modules using AgNW electrodes as both bottom and top contacts, as described in Figure 2c [99]. In these systems, AgNW bottom electrodes require planarization layers, typically ZnO thicker than ~100 nm, to suppress shunting caused by nanowire surface roughness. Fully printed semitransparent devices showed PCEs of ~4.6%, while module-scale devices (~3.5 cm^2^) achieved efficiencies of up to 6.25% with only ~0.7% absolute loss compared with small-area cells.

These results demonstrate the scalability of printed AgNW electrodes and their compatibility with monolithic module integration processes. Despite these advances, several challenges remain for printed AgNW electrodes in OSCs. A rough nanowire network can induce electrical shunts and increased recombination, particularly for bottom electrodes. In addition, the trade-off between transparency and conductivity remains a critical issue as increasing nanowire density improves conductivity but reduces optical transmission [100]. Large-area uniformity, junction resistance, and long-term mechanical stability also require further optimization [101]. Nevertheless, recent progress in interface engineering, conductive polymer integration, and nanowire junction control indicates that printed AgNW electrodes are strong candidates for next-generation flexible and large-area OSC technologies [102].

### 3.2. OSCs with Printed Polymer Electrodes

Although various conductive polymer electrodes have been developed, PEDOT:PSS is by far the most widely commercialized and practically utilized conductive polymer for printed photovoltaic electrodes. Recent advances in conductivity enhancement, interfacial engineering, and mechanical reinforcement have significantly improved the viability of PEDOT:PSS as a transparent electrode for high-performance OSCs [103]. Mechanical robustness is a key advantage of PEDOT:PSS electrodes [104]. Kim et al. demonstrated a chemically controlled transfer-printing strategy for fabricating highly conductive all-plastic PEDOT:PSS transparent electrodes compatible with flexible organic optoelectronics and printed photovoltaics [105]. The electrode fabrication process combined H_2_SO_4_ treatment with elastomeric stamp-based transfer printing, where sulfuric acid simultaneously enhanced the conductivity of PEDOT:PSS and modified its adhesion properties, enabling rapid “pick-and-place” transfer onto arbitrary substrates without damaging the underlying layers. The treated PEDOT:PSS electrodes exhibited ultrahigh electrical conductivity exceeding 4000 S cm^−1^, excellent optical transparency, and improved nanofibrillar ordering due to the removal of insulating PSS components. Flexible devices fabricated with the transferred PEDOT:PSS electrodes showed superior mechanical flexibility and stable electrical performance under repeated bending deformation. Importantly, the transfer-printing process enabled fully plastic transparent electrodes without relying on brittle ITO or vacuum deposition, highlighting the strong potential for scalable printed organic solar cells and flexible optoelectronic devices.

**Figure 3:**
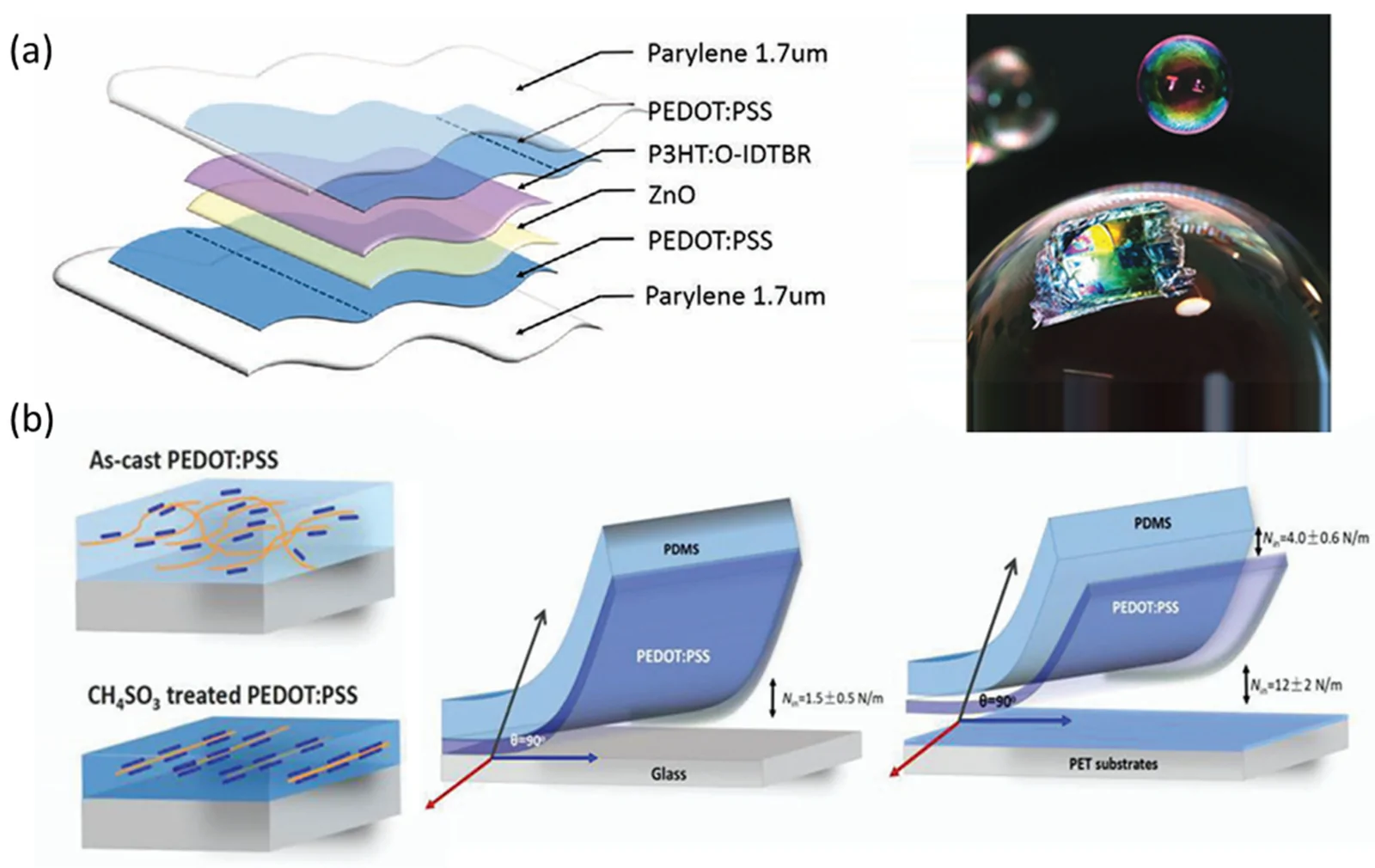
(**a**) Schematic of all the layers of the inkjet-printed OSC on a conformable parylene substrate (**left**) and photograph of the formation of ultralight inkjet-printed organic solar cells embedded in a soap bubble (**right**). Reproduced with permission from Ref. [106] Copyright 2020, John Wiley & Sons Inc. (**b**) Schematic diagram of morphology and bonding of the as-cast PEDOT:PSS and CH_4_SO_3_-treated PEDOT:PSS films and adhesion at three interfaces measured in the peeling tests. The adhesion of PEDOT:PSS is estimated using the peeling force per unit width. Reproduced with permission from Ref. [107] Copyright 2019, The Royal Society of Chemistry and the Chinese Chemical Society.

Printing-compatible PEDOT:PSS electrodes have also enabled ultrathin and fully printed OSC architectures. Bihar et al. demonstrated fully inkjet-printed, metal-free OSCs fabricated on ultrathin 1.7 µm parylene substrates using cross-linked PEDOT:PSS electrodes (Figure 3a) [106]. By introducing (3-glycidyloxypropyl)trimethoxysilane cross-linkers and post-treatment processes, the PEDOT:PSS electrodes achieved a conductivity of 360 S cm^−1^ and a sheet resistance of 27 Ω sq^−1^. The resulting ultrathin OSCs based on P3HT:O-IDTBR active layers delivered a maximum PCE of 3.61% on ultrathin substrates and 4.73% on glass substrates while exhibiting an extremely high specific power of 6.3 W g^−1^ due to the exceptionally low device weight. The encapsulated devices also retained more than 70–80% of their initial performance after exposure to water and physiological solutions, demonstrating strong potential for application in biointegrated electronics and wearable energy systems.

Another important development is transfer-printed PEDOT:PSS electrodes with enhanced interfacial adhesion and bending durability. Fan et al. employed methanesulfonic acid treatment and D-sorbitol soft interlayers to fabricate highly conductive and mechanically stable PEDOT:PSS electrodes with a figure of merit of 83 (Figure 3b) [107]. Flexible OSCs using these electrodes achieved a maximum PCE of 10.19%, outperforming conventional ITO/PET devices (8.60%). Furthermore, the devices retained approximately 92.6% of their initial efficiency after 1000 bending cycles at a bending radius of ~3 mm, while ITO-based devices showed severe degradation. Hybrid electrode architectures combining PEDOT:PSS with Ag nanowires or oxide/metal/oxide structures further improved the device efficiency to more than 10.5%, emphasizing the effectiveness of hybrid transparent electrode strategies.

Overall, printed PEDOT:PSS electrodes have demonstrated remarkable progress in terms of conductivity enhancement, mechanical flexibility, and scalable fabrication compatibility. Although challenges such as relatively lower conductivity compared with metal electrodes and moisture sensitivity remain, recent advances in chemical treatment, polymer network engineering, and hybrid electrode design indicate that PEDOT:PSS-based printed electrodes are strong candidates for next-generation flexible, wearable, and fully printed organic photovoltaic technologies.

### 3.3. OSCs with Printed Carbon Electrodes

**Figure 4:**
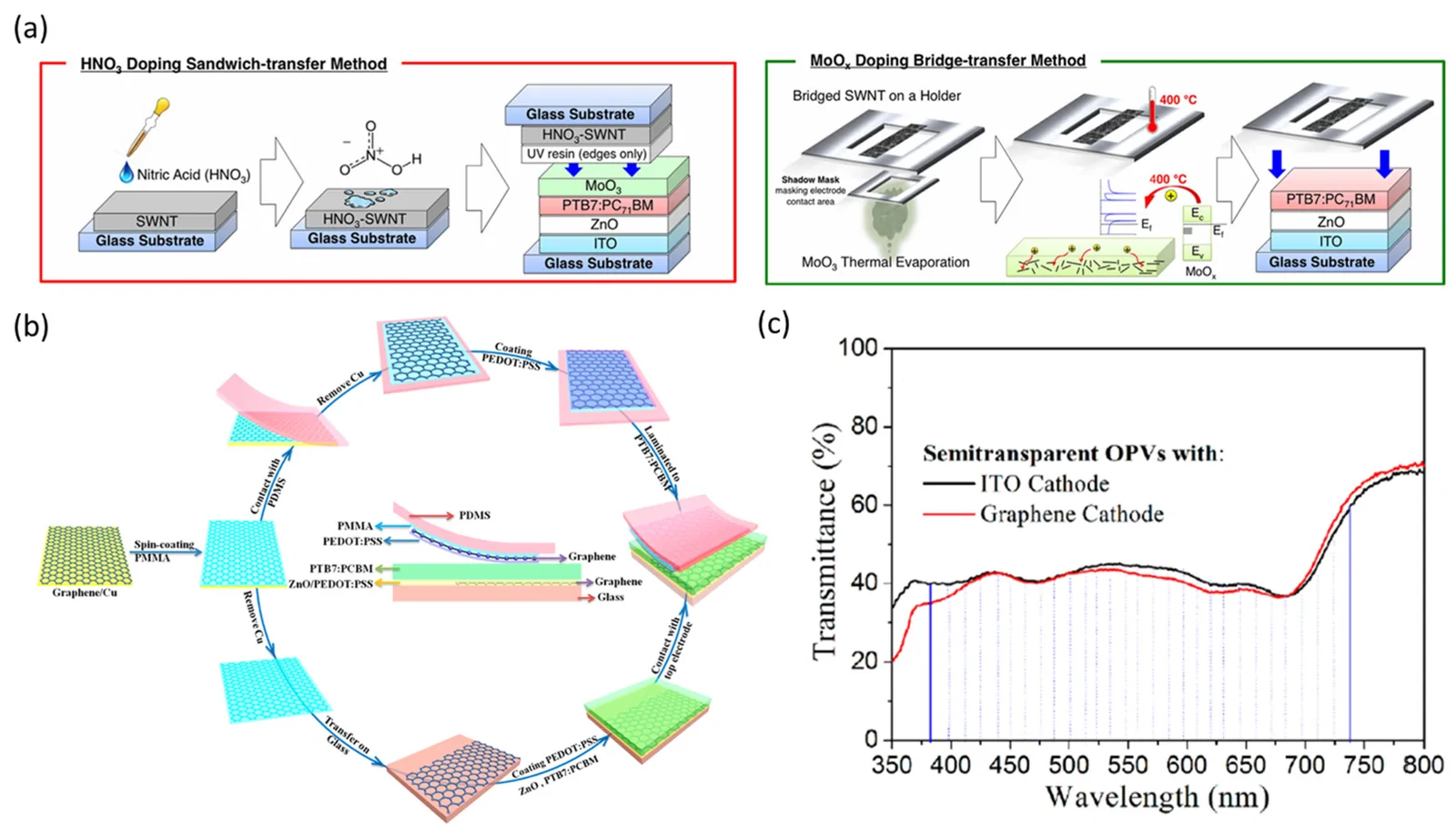
(**a**) Doping methodologies for SWCNT-laminated transparent solar cells. HNO_3_ doping sandwich transfer process (**left**) and MoO_x_ thermal doping bridge transfer process (**right**). SWCNT named as SWNT. Reproduced with permission from Ref. [108] Copyright 2016, Nature Publishing Group (**b**) Schematic diagram of the preparation procedure of OSCs with all-graphene electrodes via transfer printing. (**c**) The transmittance spectra of two semitransparent OPVs with graphene anodes and graphene or ITO cathodes. The dash lines show the visible region. Reproduced with permission from Ref. [86] Copyright 2015, American Chemical Society.

Carbon electrodes can facilitate the development of environmentally friendly, mechanically durable, and semitransparent OSCs for applications in wearable electronics, building-integrated photovoltaics, and sustainable energy-harvesting systems [66]. Carbon electrodes are typically prepared either as liquid-phase pastes or as thick films, and these forms govern the use of printing or lamination as their respective deposition methods. One of the most actively investigated carbon-based electrode materials is CNTs. Jeon et al. demonstrated metal-electrode-free transparent OSCs employing aerosol-synthesized SWCNT thin films as laminated top electrodes (Figure 4a) [108]. The freestanding SWCNT networks were fabricated by aerosol chemical vapor deposition and transferred onto OSCs through dry lamination, enabling highly transparent “window-like” devices. Undoped 90% transparent SWCNT electrodes initially yielded relatively low efficiencies (~1.8%), but novel p-doping approaches based on HNO_3_ sandwich-transfer doping and MoO_x_ bridge-transfer doping significantly improved the conductivity and interfacial charge extraction. The optimized HNO_3_-doped SWCNT devices achieved PCEs of 3.7–4.1%, with *V*_OC_ ≈ 0.70 V, *J*_SC_ ≈ 9.5 mA cm^−2^, and FF ≈ 0.65, corresponding to approximately 50% of the performance of conventional Ag-electrode reference devices, where *V*_OC_, *J*_SC_ and FF refer to open-circuit voltage, short-circuit current density and fill factor. Importantly, the CNT electrodes enabled dual-sided light harvesting and mechanically resilient transparent OSCs suitable for power-generating window applications.

Brush-painted CNT electrodes have also been explored as ultra-flexible conductive electrodes. Using a simple brush painting process directly on PET substrates under ambient conditions, Cho et al. fabricated CNT network electrodes [109]. The optimized CNT electrodes exhibited sheet resistances of approximately 200 Ω/sq with an optical transmittance of approximately 80% while maintaining extremely stable electrical properties under severe bending and stretching deformation. Flexible OSCs fabricated with these CNT electrodes achieved PCEs of up to 1.63%, while the electrodes maintained negligible resistance variation even after 10,000 bending cycles and tensile strains of up to 5.5%. This work demonstrated that mechanically compliant CNT networks can provide highly durable electrodes for flexible printed photovoltaics without relying on brittle oxide conductors.

Graphene-based electrodes have also demonstrated strong potential for semitransparent OSCs. Liu et al. reported fully carbon-based semitransparent OSCs employing graphene as both the anode and the cathode [86]. The graphene electrodes were fabricated using layer-by-layer transfer processes and further modified using PEDOT:PSS and ZnO nanoparticle interlayers to improve the conductivity and carrier selectivity, as shown in Figure 4b. After chemical modification, the resulting graphene electrodes exhibited transmittance values of approximately 94% and sheet resistances as low as 170–230 Ω/sq. Fully semitransparent all-graphene OSCs achieved PCEs of 3.06–3.35% while maintaining an average visible transparency of ~40%, demonstrating their suitability for transparent energy-harvesting applications such as smart windows and building-integrated photovoltaics (Figure 4c).

In addition to nanocarbon materials, printable graphite-based electrodes have recently been developed for environmentally sustainable OSC systems. Synkiewicz-Musialska et al. demonstrated biodegradable OSCs employing screen-printed soil-compatible carbon paste electrodes composed of graphite flakes and PEDOT:PSS binders [110]. The fully printed carbon electrodes were fabricated at low temperatures (~80 °C) on regenerated cellulose substrates and delivered PCEs of up to 2.61% under LED illumination at 1000 lx, significantly outperforming commercial carbon pastes. Importantly, the biodegradable devices exhibited acceptable mechanical durability and minimal environmental impact after long-term water exposure, highlighting the potential of carbon-based printed electrodes for transient electronics and agricultural IoT systems.

Overall, printed carbon-based electrodes provide several important advantages for OSCs, including excellent flexibility, low-temperature processability, environmental compatibility, and compatibility with R2R manufacturing. Although their electrical conductivity remains lower than that of conventional metallic electrodes in many cases, continuous advances in chemical doping, nanocarbon network engineering, hybrid electrode structures, and interface optimization are rapidly narrowing the performance gap. Consequently, printed carbon-based electrodes are expected to play an increasingly important role in next-generation flexible, transparent, lightweight, and sustainable organic photovoltaic technologies.

### 3.4. OSCs with Printed Hybrid Electrodes

**Figure 5:**
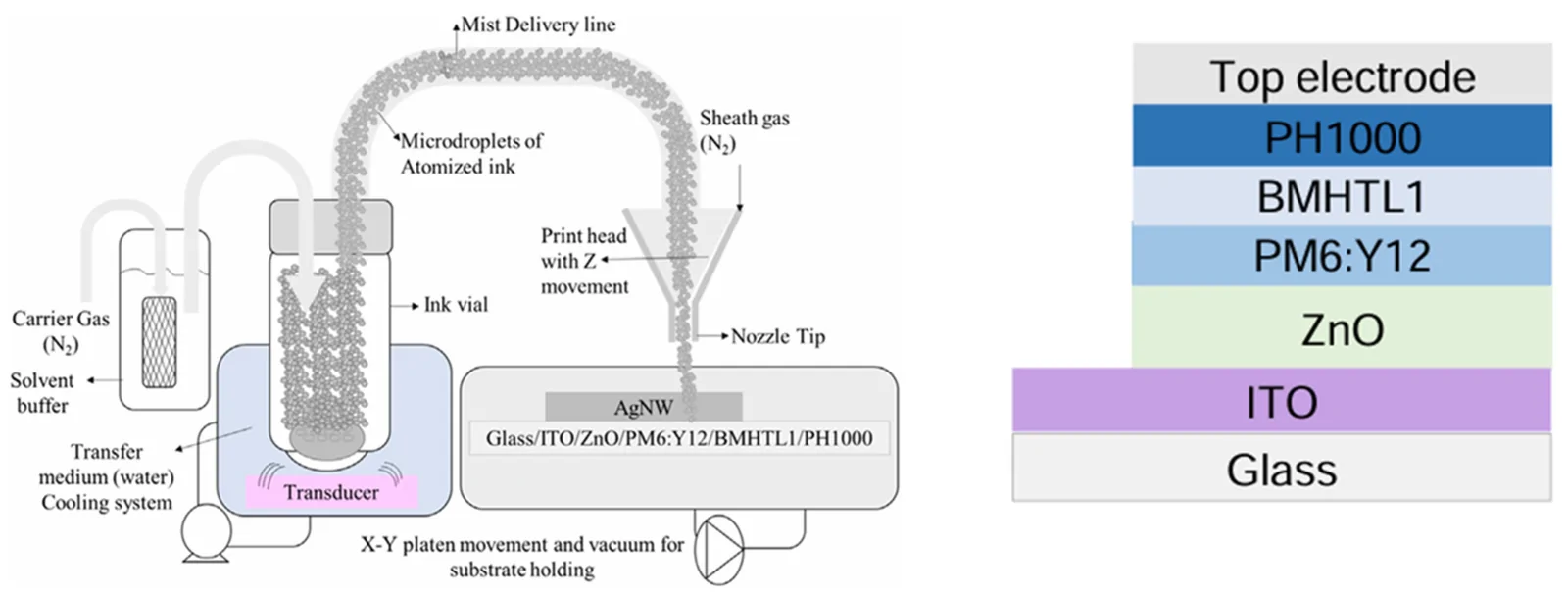
AJP working principles for AgNW ink deposition as the top electrode layer in inverted OSC devices. Reproduced with permission from Ref. [111] Copyright 2025, John Wiley & Sons Inc.

AgNWs are utilized in a hybrid form with conductive polymers such as PEDOT:PSS to resolve the surface roughness issues stemming from their one-dimensional nature. Among the various available approaches, aerosol jet printing (AJP) has attracted significant attention because of its noncontact, maskless, and high-resolution deposition capability [111]. Recent work using ultrasonic AJP to deposit AgNW top electrodes achieved PCEs of up to ~9.5%, which is among the highest reported values for printed AgNW-based OSCs (Figure 5). The transmittance of the printed AgNW electrodes ranged from ~73 to 84%, and the sheet resistance ranged from ~25 to 30 Ω sq^−1^ depending on the deposition conditions and electrode structure. In particular, introducing conductive interlayers such as PH1000 significantly reduced series resistance and improved charge collection, leading to enhanced FF and photocurrent density. These studies highlight that electrode morphology and junction connectivity are strongly influenced by printing parameters, including nozzle distance, number of printing passes, and ink flow conditions.

AgNWs are often combined with conductive polymers such as PEDOT:PSS to mitigate the surface roughness and poor interfacial contact associated with nanowire networks [112]. In this approach, AgNW/PEDOT:PSS composite electrodes supported on flexible substrates are transferred onto the active layer through thermal lamination. Devices fabricated using laminated AgNW electrodes achieved PCEs of ~2.6%, compared to ~3.4% for conventional evaporated metal electrodes. Although the efficiency remained lower because of higher series resistance and interfacial recombination, the work demonstrated the feasibility of entirely solution-processed OSC fabrication without vacuum deposition. Furthermore, the laminated electrodes exhibited excellent mechanical flexibility and stable electrical contact under repeated bending conditions.

## 4. PSCs with Printed Electrodes

### 4.1. PSCs with Printed Metallic Electrodes

Printed metallic electrodes have a significant impact on PSCs, offering high electrical conductivity, mature industrial processability, and compatibility with scalable metallization techniques. Among printable candidates, Ag paste has been the most widely considered because of its superior conductivity and facile use in PSC fabrication. However, direct deposition of metal pastes onto perovskite or organic charge transport layers presents challenges. Perovskite absorbers and organic interlayers are susceptible to degradation induced by paste solvents, chemical additives, mechanical shear during printing, and the thermal budget required for curing. Furthermore, Ag is notoriously prone to reacting with mobile halides and migrating under operational conditions such as illumination, bias, and elevated temperatures. Consequently, rather than serving as direct replacements for vacuum-evaporated Au or Ag, printed metallic electrodes in PSCs are more commonly applied as low-temperature metallization grids, auxiliary current collectors, transfer-printed contacts, or integrated components within hybrid electrode architectures.

**Figure 6:**
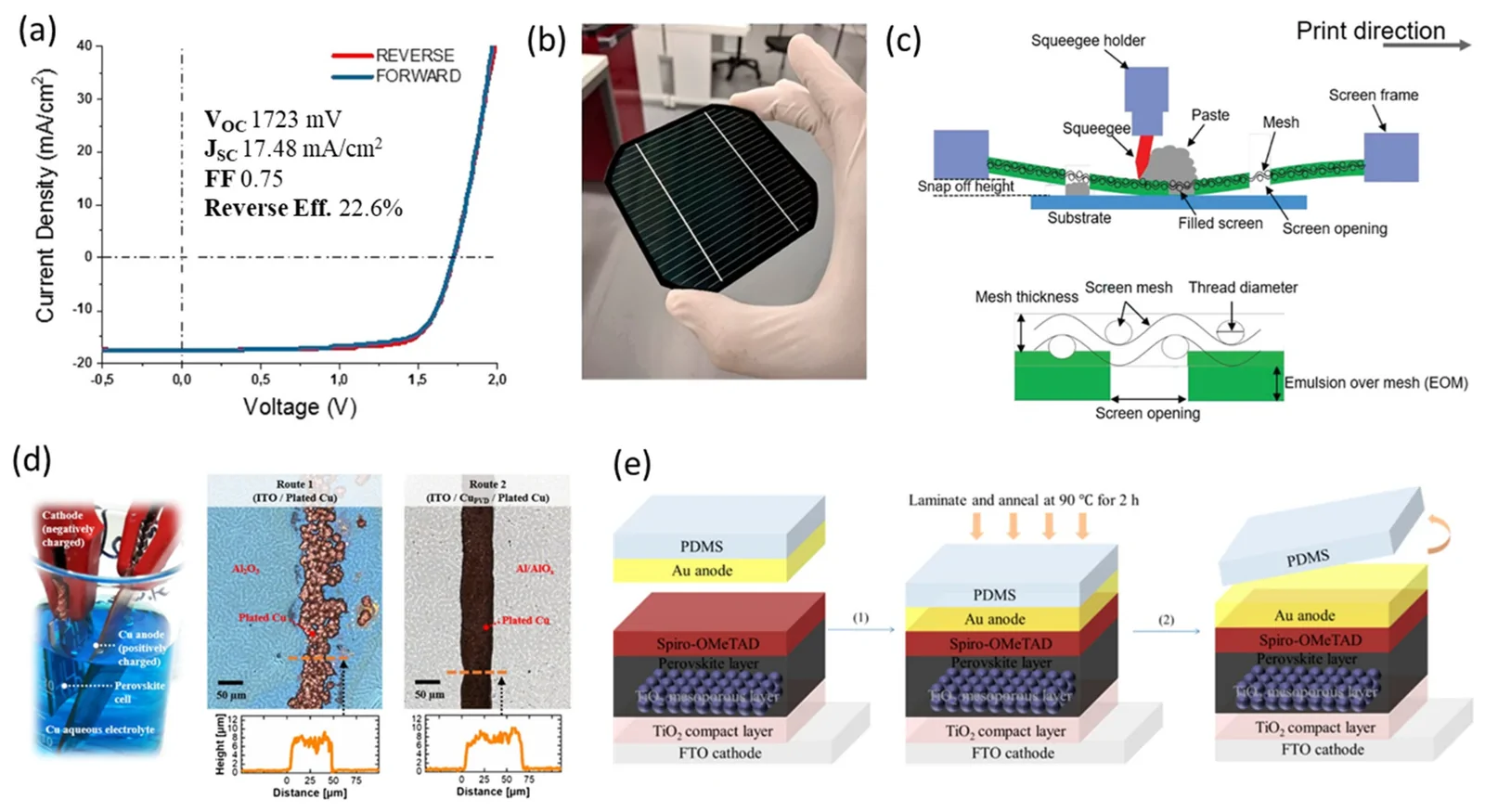
(**a**) Current–voltage curves under illumination characterization of champion large-area tandem cell. (**b**) Photo of a large-area cell after being cut down to a pseudo square shape. Reproduced with permission from Ref. [113] Copyright 2019, American Chemical Society. (**c**) Schematic of screen-printing process to realize the metallization grid (**top**) and cross-section of mesh and screen opening (**bottom**). Reproduced with permission from Ref. [114] Copyright 2023, John Wiley & Sons Inc. (**d**) Laboratory setup for copper electroplating on a perovskite solar cells (**left**). Confocal microscopy top views with respective 3D profile of plated Cu fingers metallized on perovskite substrates using two different masking routes: atomic layer deposition Al_2_O_3_ (**middle**), and physical vapor deposition Al/native AlO_x_ with underlying Cu seed on ITO (**right**). Reproduced with permission from Ref. [119] Copyright 2021, Wiley. (**e**) Fabrication procedure of transfer printing the Au top electrodes of the PSCs. Reproduced with permission from Ref. [121] Copyright 2017, American Chemical Society.

The most compelling application of printed metal pastes in PSCs has emerged in perovskite/silicon tandem solar cells. In these architectures, low-temperature metallization is crucial, as the perovskite top cell is fundamentally intolerant to the high-temperature sintering processes commonly used in crystalline silicon cells. As shown in Figure 6a,b, Kamino et al. demonstrated low-temperature screen-printed Ag metallization for two-terminal perovskite/silicon tandem solar cells, revealing that Ag paste can be successfully integrated into perovskite-containing stacks provided that the curing temperature, contact design, and grid geometry are carefully controlled [113]. Rehman et al. further explored electrode metallization for scaled perovskite/silicon tandem solar cells, emphasizing that a delicate balance among contact resistivity, transparent conducting oxide (TCO) sheet resistance, optical shading, and the thermal budget must be considered in metal-grid design (Figure 6c) [114]. Messmer et al. optimized the front TCO/metal-grid electrodes for module-integrated perovskite/silicon tandems, quantifying the optoelectronic trade-off between lateral current collection and parasitic optical loss [115]. De Rose et al. extended this scope by exploring low-temperature metallization and interconnection strategies for silicon heterojunction and perovskite/silicon tandem solar cells, including fine-line screen printing and Ag-reduced metallization concepts [116]. These studies indicate that printed metallic electrodes are particularly relevant for tandem and module architectures, where lateral resistive losses and interconnection losses strongly influence device output.

Beyond tandem structures, printed metallic grids have been strategically incorporated into single-junction PSC modules to compensate for the limited lateral conductivity of nonmetallic electrodes. Da Silva Filho et al. provided a comprehensive overview of metal grid implementation for upscaling PSCs and modules, highlighting the importance of reducing series resistance while simultaneously suppressing optical shading [117]. Raptis et al. demonstrated that integrated metallic grids can substantially increase the performance of fully printable mesoscopic PSCs by improving the conductivity of carbon electrodes [118]. In this configuration, the metal grid is intentionally decoupled from direct contact with the perovskite absorber; instead, it functions as an auxiliary current collector that supplements the printed carbon electrode. This separation of functions is important, as it enables the chemically stable carbon layer to maintain a robust interface with the perovskite stack, while the printed metal grid facilitates efficient lateral charge collection.

Alternative metallic-electrode approaches have also been explored to reduce Ag consumption and improve manufacturing compatibility. Hatt et al. demonstrated electroplated Cu contacts on PSCs by incorporating a thin protective Al_2_O_3_ masking layer, thereby validating that wet-chemical metallization can be compatible with perovskite devices, provided that the sensitive underlying layers are properly protected (Figure 6d) [119]. The authors subsequently extended electroplated Cu electrodes to two-terminal perovskite/silicon tandem solar cells, establishing Cu plating as a viable, cost-effective metallization pathway for multijunction architectures [120]. While electroplating strictly diverges from the fluidic paste printing process, it addresses similar technological challenges by forming low-resistance metal contacts under perovskite-tolerant processing conditions. Concurrently, transfer-printing techniques offer another route to circumvent direct solvent exposure and mechanical or thermal stress. As illustrated in Figure 6e, Li et al. reported fully printable organic and perovskite solar cells using transfer-printed flexible electrodes, wherein a metallic grid is prepared independently and dry-transferred onto the device stack [121]. Such a method decouples metal electrode fabrication from perovskite device assembly, thereby expanding the processing window for printable metallic contacts.

Overall, the technical limitations of printed metallic electrodes can be grouped into three coupled categories. First, paste solvents, reactive additives, printing shear, and curing temperatures can damage the perovskite absorber or organic transport layers. Second, coarse particles and nonuniform printed features increase surface roughness, which can penetrate thin functional layers, generate local shunts, and reduce the effective contact area, thereby increasing contact resistance. Third, Ag can react with mobile halides and migrate under illumination, bias, heat, or moisture, producing resistive Ag-halide phases and electrically active pathways. Accordingly, printable metallic contacts are most viable as low-temperature, finely patterned grids, protected or transfer-printed contacts, and components of hybrid architectures.

### 4.2. PSCs with Printed Polymer Electrodes

**Figure 7:**
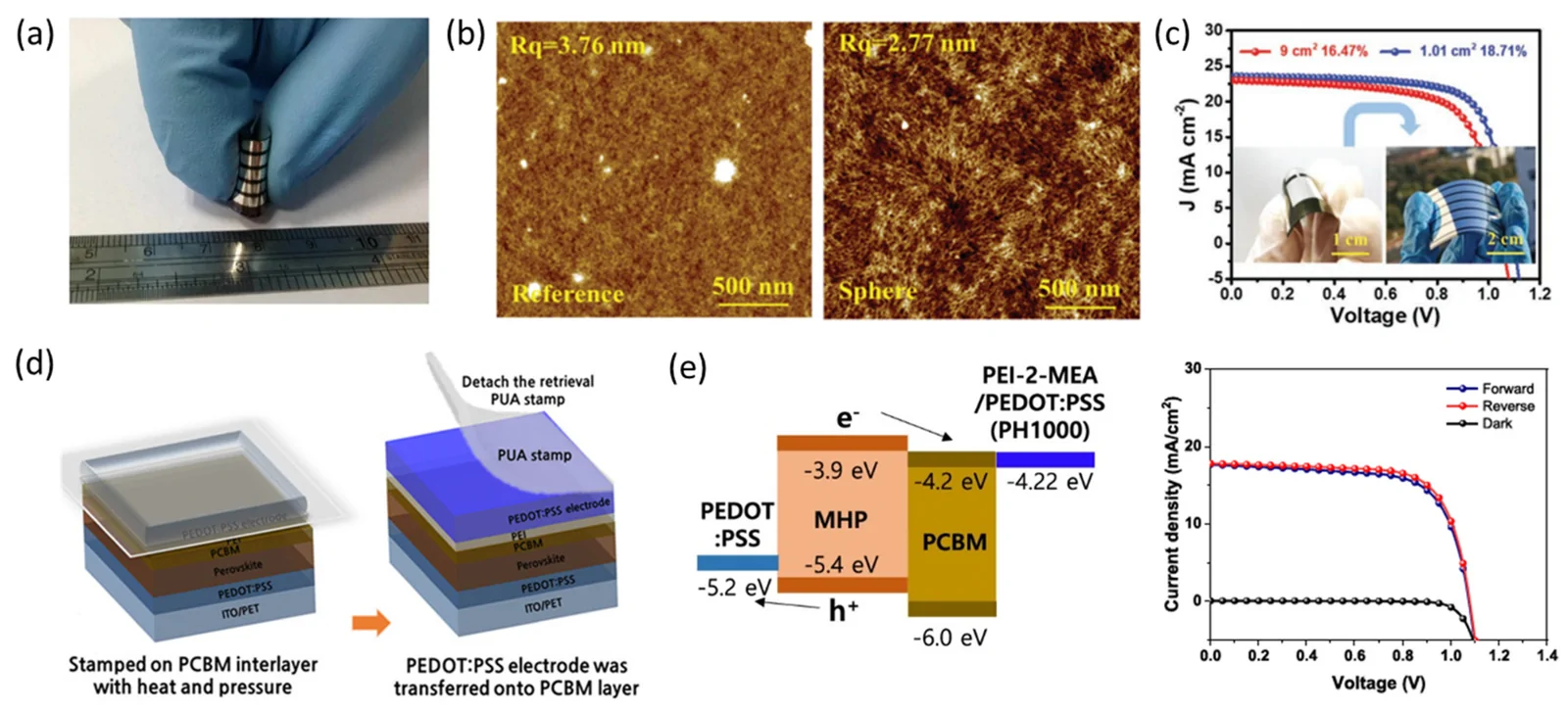
(**a**) Photo of flexible PSCs with conducting polymer PEDOT:PSS electrode. Reproduced with permission from Ref. [122] Copyright 2015, American Chemical Society. (**b**) Morphologies of transparent electrodes prepared by a conventional printing technique (left) and bionic high-speed patterned meniscus coating technique (right). (**c**) The performance of stretchable PSCs based on different device scale. Reproduced with permission from Ref. [126] Copyright 2023, John Wiley & Sons Inc. (**d**) Schematic illustration of the dry stamping transfer process of the PEDOT:PSS top electrode layer. (**e**) Energy band diagram (left) and J–V curves (right) for semitransparent flexible PSCs. Reproduced with permission from Ref. [128] Copyright 2020, American Chemical Society.

Conducting polymer electrodes provide a complementary route to printed PSCs, especially for flexible, semitransparent, and ITO-free devices. PEDOT:PSS is the prototypical conducting polymer because of its solution processability, high optical transparency, mechanical flexibility, and compatibility with low-temperature fabrication. As shown in Figure 7a, Sun et al. reported transparent conductive oxide-free PSCs using PEDOT:PSS as a transparent electrode, demonstrating a facile yet effective route toward ITO-free PSCs [122]. Vaagensmith et al. introduced plasma-treated PEDOT:PSS electrodes for ITO-free cells and reported that surface modifications can improve the properties of electrodes without relying on aggressive chemical post-treatment [123]. A major technological milestone was achieved by Hu et al., who developed a mechanically robust conducting polymer network electrode based on a printable PEDOT:PSS:CFE formulation, where CFE represents conductivity and flexibility enhancer. Through additive engineering and printing-compatible solution shearing, they obtained highly conductive and transparent polymer electrodes for efficient flexible PSCs and modules [124]. Xie et al. further investigated the mechanical stability of PEDOT:PSS-based ITO-free flexible PSCs, confirming the remarkable bending tolerance and structural resilience of polymeric contacts relative to brittle ITO-based electrodes [125]. These studies establish PEDOT:PSS as an important platform for flexible printed-electrode PSCs.

The electrical performance of the PEDOT:PSS electrode is governed by the nanoscale organization of its constituent PEDOT-rich and PSS-rich domains. In its pristine state, the polymer matrix is dominated by insulating PSS-rich regions that severely restrict charge carrier transport. Conductivity-enhancement strategies therefore aim to promote continuous and interconnected PEDOT-rich pathways. The incorporation of secondary dopants or processing additives, such as dimethyl sulfoxide (DMSO), ethylene glycol, ionic additives, and fluorosurfactants, or the application of acid treatments substantially increases electrical conductivity by promoting long-range polymeric chain ordering, selectively depleting insulating PSS, and reorganizing phase separation. Nevertheless, the application of these modifying agents within PSC architectures necessitates stringent precision, as aggressive chemical modifications can detrimentally alter the work function, exacerbate surface roughness, introduce deleterious hygroscopic residues, or directly degrade the underlying perovskite and transport interlayers.

Consequently, the development of high-performance polymer electrodes relies on the simultaneous optimization of sheet resistance, optical transmittance, interfacial wetting, energetic alignment, and environmental durability. Illustrating this design approach, Gong and coworkers pioneered a bionic, high-speed patterned meniscus printing technique to precisely align the network geometry of a PEDOT:PSS electrode on a flexible hydroxypropyl methylcellulose (HPMC) substrate (Figure 7b) [126]. By combining the novel printing method with a Zn(TFSI)_2_ additive, a highly oriented morphology was obtained that concurrently enhanced optical transmittance and lateral conductivity, resulting in a 20.04% PCE (1 cm^2^) in stretchable PSCs (Figure 7c). Given its ease of processing and high versatility, PEDOT:PSS has attracted substantial interest as a transparent top electrode for semitransparent or metal-electrode-free PSCs. Jiang et al. demonstrated metal-electrode-free PSCs with transfer-laminated PEDOT:PSS top electrodes, avoiding direct coating of aqueous PEDOT:PSS onto the perovskite layer [127]. This approach is particularly advantageous because it circumvents solvent incompatibility issues while enabling a transparent top contact. Lee et al. subsequently reported a reproducible dry-stamping transfer process to integrate PEDOT:PSS transparent top electrodes into flexible, semitransparent PSCs, further confirming that polymer electrodes can be integrated through transfer processes rather than direct deposition (Figure 7d,e) [128]. Such transfer-mediated strategies are particularly useful when polymeric inks are deployed atop highly solvent-sensitive perovskite or organic layers.

Despite these advantageous features, PEDOT:PSS electrodes still face significant limitations. Even conductivity-enhanced formulations typically exhibit elevated sheet resistance relative to standard ITO or printed metal grids, which restricts efficient current collection over large-area module dimensions. In addition, the acidic and hygroscopic nature of the PSS domain possibly can accelerate the degradation of contiguous functional layers in the absence of proper interfacial modification or robust encapsulation. Polymeric electrodes also induce parasitic absorption as the film thickness increases, imposing an inherent trade-off between sheet resistance and optical transmittance. Consequently, PEDOT:PSS electrodes are optimally deployed in flexible or semitransparent PSCs when strategically combined with additional conductive networks and processed via advanced lamination methods.

### 4.3. PSCs with Printed Carbon Electrodes

**Figure 8:**
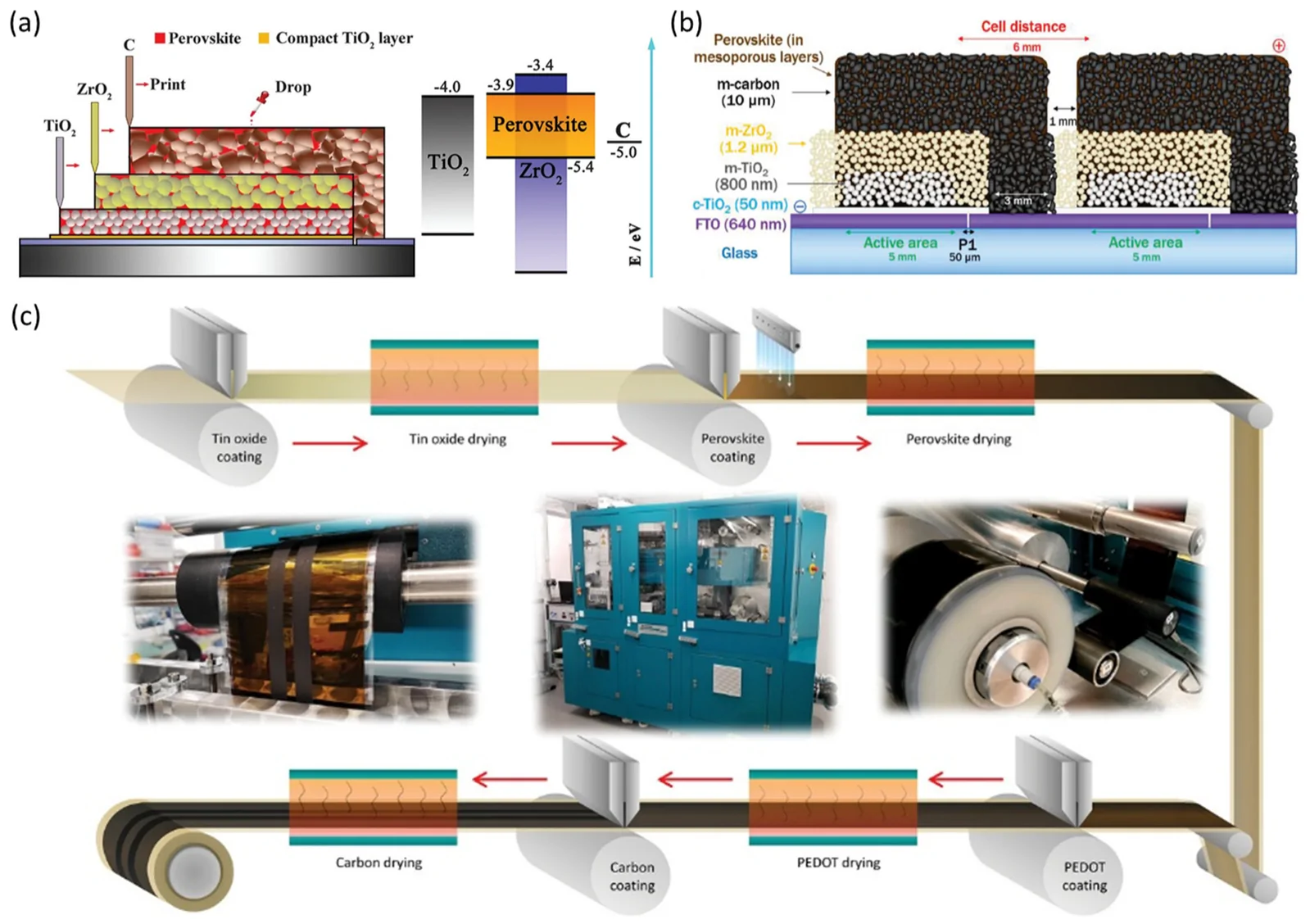
(**a**) Schematic drawing showing the cross section of the triple-layer perovskite-based fully printable mesoscopic solar cell. C stands for carbon as an electrode (**left**) and corresponding energy band diagram (**right**). Reproduced with permission from Ref. [129] Copyright 2014, American Association for the Advancement of Science. (**b**) Cross-section schematics of adjacent cells in the module with nominal thickness of each layer, highlighting the laser-etched FTO, patterning of TiO_2_ blocking layer and the electrical vertical connection, ensured by the carbon back contact. Reproduced with permission from Ref. [133] Copyright 2018, John Wiley & Sons Inc. (**c**) Schematic visualization of roll-to-roll slot-die coating method for fabrication of perovskite solar cells with carbon top electrode. Reproduced with permission from Ref. [140] Copyright 2023, John Wiley & Sons Inc.

Printed carbon electrodes represent the most comprehensively investigated class of vacuum-free contacts for PSCs. Carbon materials possess profound industrial advantages; they are inherently cost-effective, chemically inert, highly hydrophobic, and Earth-abundant while exhibiting exceptional compatibility with scalable, high-throughput printing or coating methods such as screen printing, doctor blading, bar coating, and spray coating. Compared with thermally evaporated metals (e.g., Al or Ag), carbon is less prone to halide-mediated corrosion and metal-ion migration. As a result, carbon electrodes have been extensively evaluated as robust alternatives to conventional back electrodes, particularly within hole transport material-free configurations, fully printable mesoscopic scaffolds, and long-term stable module architectures. As shown in Figure 8a, a milestone development was the hole-conductor-free, fully printable mesoscopic PSC reported by Mei et al. [129]. This device featured a TiO_2_/ZrO_2_/carbon triple mesoscopic scaffold, which served as a porous template for seamless capillary infiltration of the perovskite precursor solution. In such an architecture, the carbon electrode is not simply a rear contact; it is part of the printed mesoscopic scaffold that influences perovskite infiltration, crystallization, charge extraction, and device stability. Furthermore, on the basis of this design, Liu et al. improved mesoscopic carbon-based PSCs by introducing an organosilane self-assembled monolayer to modify interfacial energetics and, thus, carrier dynamics [130]. Similarly, Zhang et al. investigated the morphological properties of carbon counter electrodes and reported that the carbon particle size, size distribution, film thickness, bulk conductivity, and sheet resistance strongly influence the photovoltaic parameters [131]. These studies established that printed carbon electrodes should be engineered together with the perovskite infiltration process and interfacial structure.

The translation of carbon-electrode technologies into large-area modules reflects the industrial scalability of these vacuum-free architectures. Priyadarshi et al. reported a large-area monolithic perovskite solar module with a 70 cm^2^ active area based on a printable mesoscopic carbon matrix [132]. As illustrated in Figure 8b, De Rossi et al. subsequently demonstrated all-printable perovskite solar modules with a 198 cm^2^ active area, thereby validating the extensibility of screen-printable carbon architectures to module-scale fabrication [133]. These module studies are significant in that they highlight both the potential of printed carbon electrodes for scalable fabrication and the remaining challenges. Carbon electrodes enable vacuum-free and thermally stable module fabrication, but the cell-to-module efficiency roll-off remains intricately tied to the bulk sheet resistance of the carbon formulation, geometric interconnection designs, macroscopic uniformity of perovskite infiltration, and overall quality of laser scribing lines.

To expand the structural and processing versatility of vacuum-free devices, low-temperature carbon electrodes have been intensively developed. The conventional high-temperature sintering (≥400 °C) required for mesoscopic carbon scaffolds is fundamentally incompatible with soft polymer substrates or underlying organic charge transport layers that possess a strict thermal budget. To address this material incompatibility, Zhou et al. successfully constructed hole-conductor-free and metal-electrode-free TiO_2_/MAPbI_3_ heterojunction PSCs using a low-temperature-processed carbon counter electrode [134]. Similarly, Yang et al. introduced an all-carbon counter electrode configuration comprising a mesoscopic carbon film integrated with a flexible graphite sheet, which was specifically tailored to enhance hole extraction in hole-conductor-free PSCs [135]. Furthermore, Jiang et al. systematically optimized the binder and solvent chemistry of carbon pastes to yield highly conductive, perovskite-compatible formulations cured at low temperatures, enabling fully printable PSCs with improved lateral charge transport [136]. In a comprehensive overview, Bogachuk et al. reported that shifting toward a low-temperature carbon processing paradigm significantly broadens the manufacturing window, unlocking compatibility with planar topologies, flexible plastics, and solution-deposited charge [137].

Planar carbon-electrode PSCs represent an important step toward simplified device fabrication. In planar devices, a carbon formulation is directly deposited on top of a perovskite/charge transport layer stack, replacing the vacuum-evaporated metal electrodes typical of conventional architectures. However, direct coating of carbon paste possibly damages the underlying perovskite or organic transport layer because of detrimental solvent penetration, residual insulating binders, interface roughness, and induced mechanical stress. In addition, suboptimal physical adhesion and poor interfacial contact between the carbon layer and the contiguous semiconductor frequently increase the contact resistance and, thus, strongly decrease the FF. Du et al. mitigated such interfacial deficits by introducing bilayer hole-transporting contacts in fully printed carbon-electrode PSCs, demonstrating that decent interfacial engineering can suppress nonradiative charge recombination and improve charge extraction at the carbon contact [138]. Similarly, Iqbal et al. reported fully printed HTL-free MAPbI_3_ PSCs with low-temperature-printed carbon electrodes, further supporting the potential of carbon electrodes for simplified, low-cost device structures [139].

Advancing toward industrial commercialization, recent R2R processing studies have emphasized the profound viability of printed carbon electrodes. As shown in Figure 8c, Beynon et al. reported fully R2R-printed perovskite photovoltaics enabled by a solution-processed carbon formulation, successfully substituting for vacuum-evaporated metallic back contacts within a continuous pilot-scale processing line [140]. Weerasinghe et al. expanded this high-throughput platform by fabricating entire PSC modules via continuous R2R printing under ambient conditions, strategically coupling printed carbon electrodes with screen-printed Ag grids to facilitate rapid charge extraction [141]. These demonstrations show that carbon electrodes are highly attractive for vacuum-free manufacturing but suggest that carbon alone may not be able to provide sufficient lateral conductivity for large-area modules. Consequently, contemporary module structures increasingly hybridize printed carbon matrices with auxiliary metallic current-collecting grids or prefabricated laminated conductive contacts to concurrently achieve stability and low series resistance.

Overall, printed carbon electrodes exhibit exceptional convergence of low material cost, chemical robustness, hydrophobicity, and scalable processability. However, their considerable potential is often limited by inferior bulk conductivity compared with metals, imperfect interfacial contact, solvent orthogonality issues, and residual binder effects after low-temperature curing. Overcoming these technical barriers to realizing high-performance PSCs demands a coordinated, multidimensional optimization approach encompassing carbon paste formulation, particle morphology, electrode thickness, solvent systems, curing temperatures, interfacial passivation layers, and module-level current collection geometry. Ultimately, carbon electrodes should be regarded not merely as cost-effective metal substitutes but also as key components that define the structure of stable and printable PSCs.

### 4.4. PSCs with Printed Hybrid Electrodes

**Figure 9:**
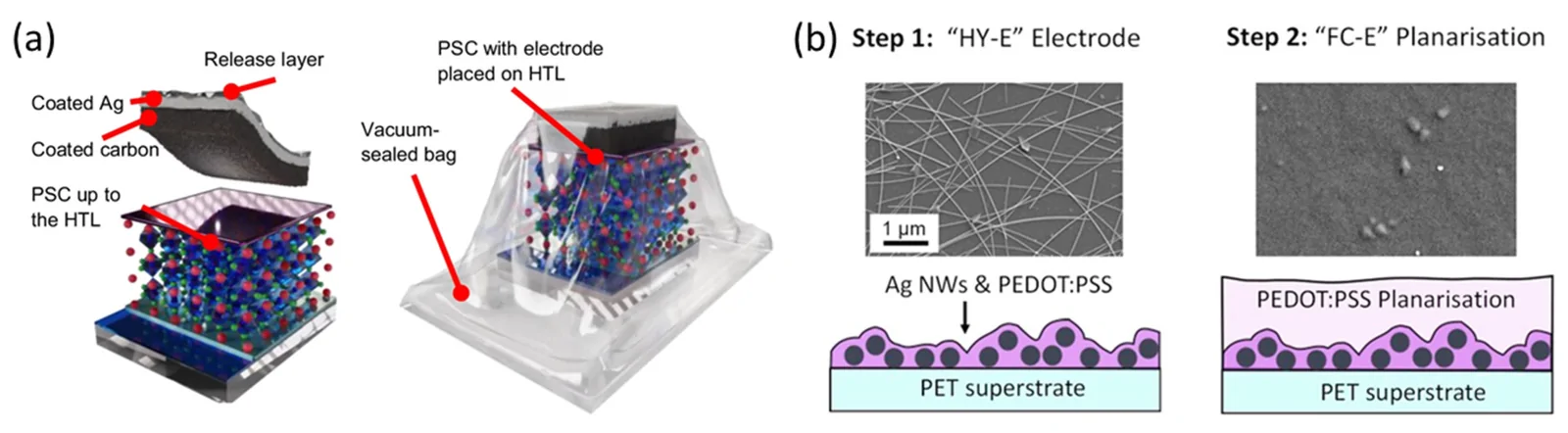
(**a**) Perovskite solar cell device stack fabricated up to the HTL and the coated bilayer electrode on a release layer, and vacuum sealing of the PSC stack and electrode. Reproduced with permission from Ref. [142] Copyright 2024, Nature Publishing Group. (**b**) Schematic of the two step approach used. First, a Clevios HY-E formulation was slot-die coated to form a PEDOT:PSS transparent electrode. In the second step, a Clevios FC-E formulation was slot-die coated on top to form a ∼250 nm thick PEDOT:PSS planarization layer. The SEM images are of an HY-E electrode (10 μL/min, 8.3 μm wet film) before and after planarization (same scale bar for both). Reproduced with permission from Ref. [143] Copyright 2017, John Wiley & Sons Inc.

Hybridization strategies have garnered widespread research attention in the development of printed contacts, typically manifesting as metal/carbon, metal/conducting-polymer, or polymer/carbon composite configurations. Sutherland et al. developed vacuum- and solvent-free Ag/carbon bilayer electrodes for R2R-fabricated PSCs, utilizing an ex situ fabrication approach in which the entire electrode structure is prepared separately and transferred onto the device stack [89]. This dry-transfer strategy effectively shields the underlying active perovskite layer from aggressive solvent exposure while synergistically combining the high conductivity of Ag with the chemical stability of carbon. In a subsequent study, the authors introduced high-pressure isostatic lamination for carbon electrode-based PSCs, demonstrating that laminated hybrid networks substantially improve physical interface contact and continuous processing compatibility (Figure 9a) [142]. These studies highlight a key advantage of laminated hybrid configurations: the complete decoupling of electrode optimization from the thermal and chemical constraints of the active perovskite stack, permitting seamless integration under benign, nondestructive conditions.

PEDOT:PSS/metal hybrid transparent electrodes are another promising class of hybrid contacts for ITO-free and flexible PSCs. In these configurations, the polymeric matrix contributes to macroscopic film uniformity, flexibility, work-function tunability, and conformal contact formation, while embedded AgNWs or metal grids drastically reduce sheet resistance. Sears et al. successfully developed ITO-free flexible PSCs using R2R slot-die-coated AgNW meshes, proving that such nanostructured transparent conductors can be integrated into flexible perovskite devices via scalable processing (Figure 9b) [143]. These efforts show that polymer/metal hybrid electrodes represent a promising platform for flexible and semitransparent PSCs, particularly when optical transparency, lateral conductivity, mechanical compliance, and low-temperature budgets should be simultaneously satisfied.

The foremost challenge hindering the widespread deployment of hybrid printed electrodes lies in their inherent interfacial complexity. Each constituent material introduces new interfaces, possible chemical reactions, optical losses, adhesion problems, and mechanical failure pathways. For instance, AgNWs are susceptible to corrosion in halide-containing environments, PEDOT:PSS introduces detrimental acidity and hygroscopic moisture sensitivity, carbon layers frequently suffer from poor physical contact, and metal grids can induce local shunts when deposited atop rough or mechanically fragile underlying layers. Thus, successful hybrid electrode design necessitates careful control of the buffer layers, solvent orthogonality, surface roughness, lamination pressure, work-function alignment, and encapsulation. Despite these challenges, hybrid printed electrodes are likely to be among the most viable routes toward the production of printed PSCs, offering the unique capacity to decouple and individually optimize charge transport dynamics, optical transmittance, and structural flexibility without sacrificing chemical compatibility with the active perovskite matrix.

## 5. Summary

**Figure 10:**
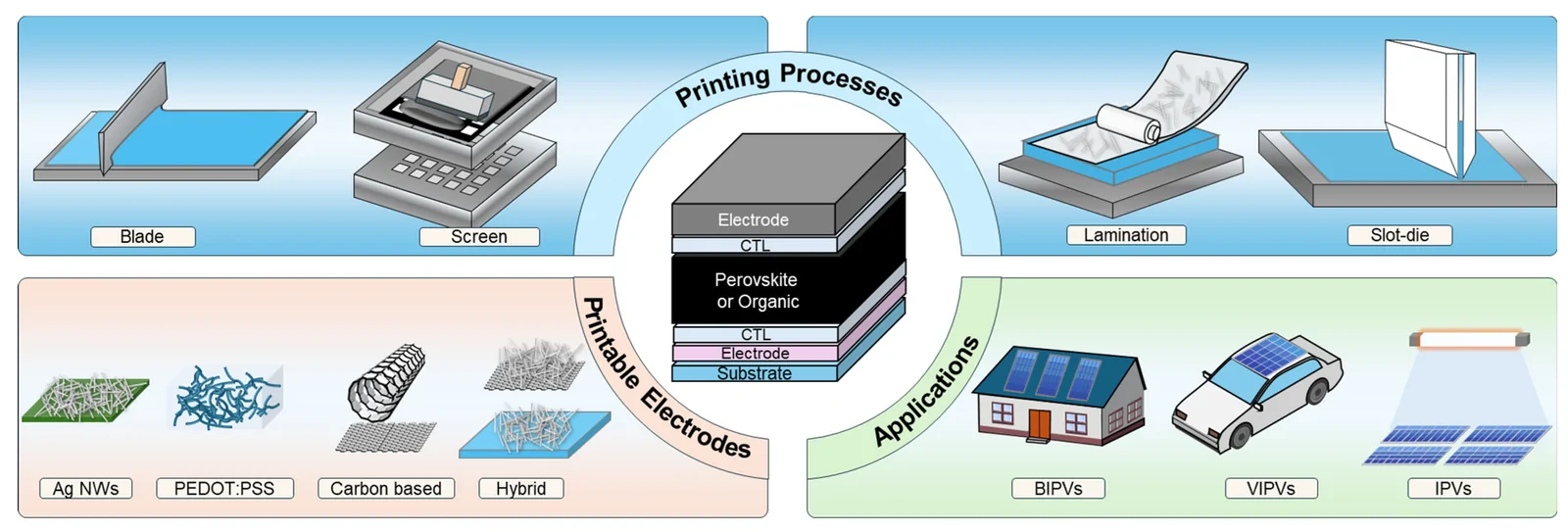
Schematic illustration of various printing processes, printable electrodes, and their applications. (IPVs: indoor photovoltaics).

In summary, we reviewed printable electrode materials and their applications in OSCs and PSCs using various printing methods (Figure 10). The photovoltaic parameters of representative solar cells discussed in this review are summarized in Table 1 for OSCs and Table 2 for PSCs. Paste-based electrodes such as metallic paste and carbon paste represent the most viable pathway for opaque solar cells, while PEDOT-based hybrids hold greater potential for semitransparent photovoltaics. For the tandem solar cells featuring a silicon bottom cell, transparent conductive electrodes such as ITO are essential for the top electrode; thus, paste-based electrodes printable in grid patterns hold significant promise. Additionally, printed electrodes are inherently advantageous for flexible applications in contrast to conventional vacuum-deposited electrodes.

Among metallic electrodes, Ag paste and AgNWs are utilized due to their low-temperature sintering processes. Ag paste has been widely used since the early development of silicon solar cells and has been employed as a grid-type electrode on ITO electrodes, thereby effectively reducing the ohmic loss of the ITO layer. Among polymer electrodes, several conductive polymers, such as PANI, PPy, and PEDOT:PSS, exhibit conductivities approaching 1000 S/cm. Among these conductive polymers, PEDOT:PSS has been widely used because it is water-dispersible and has suitable viscosity for printing processes. In addition to metallic and polymer electrodes, carbon electrodes have attracted considerable attention, and various types of carbon electrodes have been employed in different forms because of their distinct characteristics depending on the carbon material used. Hybrid-type electrodes have also been developed, with AgNW/PEDOT:PSS and Ag paste/carbon electrodes being representative examples.

Ultimately, the selection of a printed electrode is not a matter of identifying a single ideal candidate, but, rather, matching material trade-offs to the specific device architecture and operational demands. Metallic inks excel in high-throughput grid topologies owing to their unmatched conductivity, yet concerns regarding silver consumption, interface roughness, and chemical damage impede their direct deployment as continuous planar contacts. Conversely, PEDOT:PSS and related polymeric formulations offer the optical transparency and mechanical elasticity required for flexible or semitransparent form factors, despite being bound by modest intrinsic conductivity and environmental fragility. For opaque back contacts, carbon-based pastes provide an exceptionally resilient, low-cost solution tailored for vacuum-free fabrication, though their performance remains sensitive to interparticle resistance and binder kinetics. From this perspective, hybrid electrode configurations emerge as a pragmatic compromise for large-area modules, seamlessly pairing a chemically inert interfacial layer with an auxiliary conductive grid to bridge the gap between stability and charge-collection efficiency.

Among the various printing methods, screen-printing processes are widely employed for top electrodes in solar cells because they are well suited for coating the high-viscosity inks required to achieve low sheet resistance. In the case of PEDOT:PSS, PH1000 is generally utilized as a transparent electrode in hybrid electrodes or semitransparent solar cells rather than as a standalone electrode. Thus, solutions with viscosities of approximately 50 mPa·s can be processed using blade coating or slot-die coating techniques. In addition, lamination processes have been employed for the fabrication of sheet-type carbon electrodes. However, their performance remains inferior to that of evaporated metal electrodes, and the reproducibility in large-area modules is still insufficient. Although Ag grids are effectively employed in tandem solar cells that require top ITO electrodes and for reducing the sheet resistance of bottom ITO electrodes, the use of expensive ITO electrodes remains unavoidable. Therefore, the development of printable electrodes and their associated processing technologies is needed to reduce the cost of OSCs and PSCs.

## Figures and Tables

**Table 1:** Photovoltaic parameters of OSCs with printed electrodes.

Device Configuration	Electrode	Voc (V)	Jsc (mA/cm^2^)	FF (%)	PCE (%)	Ref.	Year
Ag/ZnO/PV2000/PEDOT:PSS/Ag	Inkjet-printed Ag	0.77	10.4	51.0	4.1	[94]	2015
ITO/ZnO/P3HT:PCBM/PEDOT:PSS/Ag	Inkjet-printed Ag	0.557	9.2	61.4	3.2	[95]	2016
ITO/ZnO/P3HT:PCBM/PEDOT:PSS/Ag	Screen-printed Ag	0.60	7.90	54.3	2.58	[96]	2015
ITO/PEDOT:PSS/D18:Y6/PDIN/FM	Blade-coated FM	0.839	25.58	74.86	16.07	[98]	2023
Ag/Al-doped ZnO/P3HT:PCBM/PEDOT:PSS/AgNW	Doctor-bladed Ag/Spray-coated AgNW	0.564	7.52	59.3	2.50	[102]	2015
AgNW:ZnO/PV2000:PCBM/PEDOT:PSS/AgNW	Doctor-bladed AgNW	0.73	13.27	50	4.88	[99]	2021
H_2_SO_4_-treated PEDOT:PSS/PTB7-Th:PC_71_BM/Ca/Al	Transfer-printed PEDOT:PSS	0.77	14.22	70	7.7	[105]	2015
PEDOT:PSS/ZnO/P3HT:O-IDTBR/PEDOT:PSS	Inkjet-printed PEDOT:PSS	0.71	10.39	64.4	4.73	[106]	2020
AgNW/CH_4_SO_3_-treated PEDOT:PSS/PEDOT:PSS/PBDB-T:IT-M/PDINO/Al	Transfer-printed PEDOT:PSS w/AgNW	0.91	16.95	66.2	10.21	[107]	2019
ZnO/Ag/ZnO/CH_4_SO_3_-treated PEDOT:PSS/PEDOT:PSS/PBDB-T:IT-M/PDINO/Al	Transfer-printed PEDOT:PSS w/ZnO/Ag/ZnO	0.92	17.36	66.0	10.55	[107]	2019
ITO/ZnO/PTB7:PC_71_BM/MoO_x_/CNT	Transfer-printed CNT	0.70	9.0	65	4.1	[108]	2016
CNT/PEDOT:PSS/P3HT:PCBM/Ca/Al	Brush-printed CNT	0.566	7.12	40.4	1.632	[109]	2014
Graphene/PEDOT:PSS/ZnO/PTB7:PCBM/MoO_x_/Ag	Transferred graphene	0.725	14.1	52.8	5.64	[86]	2015
ITO/ZnO/P3HT:PCBM/PEDOT:PSS/Soil-compatible carbon paste	Screen-printed carbon	0.34	0.056	32	1.98	[110]	2025
ITO/ZnO/PM6:Y12/BMHTL1/PH1000/AgNW	Aerosol-jet-printed AgNW	0.767	18.4	60.8	8.57	[111]	2025
ITO/P3HT:PCBM/AgNW	Laminated AgNW	0.605	7.78	54	2.53	[112]	2021

**Table 2:** Photovoltaic parameters of PSCs with printed electrodes.

Device Configuration	Electrode	Voc (V)	Jsc (mA/cm^2^)	FF (%)	PCE (%)	Ref.	Year
Ag/ITO/a-Si:H(p)/a-Si:H(i)/cSi/a-Si:H(i)/a-Si:H(n)/ITO/NiO/perovskite/C_60_/SnO_2_/ITO/Ag(MgF_2_)	Screen-printed Ag paste grid	1.723	17.48	75	22.6	[113]	2019
ITO/PTAA/PFN/perovskite/C_60_/SnO_x_/ITO/Al_2_O_3_/Cu grid	Electroplated Cu	1.05	14.6	73.7	11.3	[119]	2021
Ag/ITO/a-Si:H(p)/a-Si:H(i)/p-type c-Si/a-Si:H(i)/a-Si:H(n)/ITO/Spiro-TTB/Perovskite/C_60_/SnO_2_/ITO	Screen-printed Ag + Plated Cu	1.764	13.4	62.8	14.9	[120]	2022
FTO/c-TiO_2_/m-TiO_2_/perovskite/Spiro-OMeTAD/Au	Transfer-printed Au	1.05	20.74	63	13.72	[121]	2017
Glass/PEDOT:PSS/PEDOT:PSS/perovskite/PCBM/Al	MSA-treated PEDOT:PSS	0.97	17.4	65	11.0	[122]	2015
Glass/PEDOT:PSS/PEDOT:PSS/Perovskite/PCBM/Al	Plasma-treated PEDOT:PSS	0.81	19.6	66	10.5	[123]	2017
PET/PEDOT:PSS:CFE/PEDOT:PSS/Perovskite/PCBM/Ag	Slot-die printing of PEDOT:PSS:CFE network	1.08	22.38	79	19.0	[124]	2019
(HPMC)/hc-PEDOT:PSS/SnO_2_/perovskite/Spiro-OMeTAD/Ag	Patterned meniscus coating of PEDOT:PSS	1.15	23.16	75.25	20.04	[126]	2023
FTO/c-TiO_2_/m-TiO_2_/perovskite/spiro-OMeTAD/PEDOT:PSS	Transfer-laminated PEDOT:PSS	0.97	16.40	57	9.05	[127]	2015
PET/ITO/PEDOT:PSS/MHP/PCBM/PEDOT:PSS	Dry-stamped PEDOT:PSS top electrode	1.07	17.7	71.7	13.6	[128]	2020
FTO/c-TiO_2_/m-TiO_2_/m-ZrO_2_/perovskite/carbon	Screen-printed carbon electrode	0.858	22.8	66	12.84	[129]	2014
FTO/c-TiO_2_/m-TiO_2_/m-ZrO_2_/perovskite/carbon	Screen-printed carbon electrode	0.872	19.6	75	12.7	[130]	2015
FTO/c-TiO_2_/m-TiO_2_/m-ZrO_2_/perovskite/carbon	Screen-printed carbon electrode	0.894	18.06	72	11.65	[131]	2015
FTO/TiO_2_/ZrO_2_/carbon/perovskite (module)	Screen-printed carbon electrode	9.63	17.72	62.9	10.75	[132]	2016
FTO/c-TiO_2_/m-TiO_2_/m-ZrO_2_/perovskite/carbon (module)	Screen-printed carbon electrode	19.7	0.98	34.2	6.6	[133]	2018
FTO/TiO_2_/perovskite/carbon	doctor-blade-printed carbon electrode	0.8	21.02	54	9.08	[134]	2014
FTO/TiO_2_/perovskite/carbon/graphite sheet	Screen-printed carbon electrode	0.953	18.73	57.2	10.2	[135]	2014
FTO/c-TiO_2_/m-TiO_2_/m-ZrO_2_/perovskite/carbon	Screen-printed carbon electrode	0.88	23.12	69	14.04	[136]	2018
Glass/ITO/SnO_2_/perovskite/TaTm/P3HT/carbon	blade-printed carbon electrode	1.11	23.7	76	19.2	[138]	2023
ITO/SnO_2_/perovskite/carbon	Screen-printed carbon electrode	1.02	22.77	61	14.17	[139]	2023
ITO/SnO_2_/perovskite/carbon	stencil-coated carbon electrode	n/a	n/a	n/a	10.8	[140]	2023
PET/TCE/ITO/SnO_2_/perovskite/carbon/Ag grid	Screen-printed carbon + Ag grid	1.02	19.9	76.1	15.5	[141]	2024
ITO/SnO_2_/perovskite/Spiro-OMeTAD/carbon/Ag	laminated carbon/Ag electrode	1.10	23.2	81.3	20.8	[142]	2024
PET/AgNW-PEDOT:PSS/PCBM/Al	Slot-die-coated AgNW/PEDOT:PSS electrode	n/a	n/a	n/a	11.0	[143]	2017

## Data Availability

No new data were created or analyzed in this study. Data sharing is not applicable to this article.
